# An Anatomical Intraradicular Retainer Fabricated Using a Putty Index: A Case Report

**DOI:** 10.7759/cureus.109154

**Published:** 2026-05-18

**Authors:** Shruthi Venkateswaran, Aravindhan Venkatapathi, A Senthilkumar, Kathiravan I

**Affiliations:** 1 Conservative Dentistry and Endodontics, JKKN Dental College and Hospital, Namakkal, IND; 2 Conservative Dentistry and Endodontics, Sri Balaji Vidyapeeth (Deemed to be University), Pondicherry, IND

**Keywords:** aesthetic, anatomical post, endodontically, favorable, fiber post, masticatory, microleakage, prognosis, rehabilitation, root fracture

## Abstract

Post-endodontic restoration is a complex process that requires careful consideration of multiple factors, with the primary goal of restoring function, esthetics, and structural integrity while preventing microleakage and root fractures. The choice of restorative approach depends largely on the remaining tooth structure. In the present case, a severely weakened endodontically treated tooth with significant loss of radicular dentin was rehabilitated using a customized anatomic fiber post technique. After completion of root canal therapy, the post space was prepared, and a fiber post was relined with composite resin to closely adapt to the canal morphology, ensuring optimal fit and stress distribution. This was followed by core buildup and definitive full-coverage restoration, resulting in satisfactory functional and esthetic outcomes. The use of anatomic posts demonstrated a favorable prognosis, particularly in fragile roots, by enhancing retention, minimizing cement thickness, and reducing the risk of root fracture. This case highlights that individualized post adaptation is superior to conventional prefabricated posts in compromised canals, emphasizing the importance of preserving remaining dentin, achieving proper post adaptation, and selecting materials that mimic the biomechanical properties of natural tooth structure as key learning points for long-term success.

## Introduction

Rehabilitation of severely damaged endodontically treated teeth is a common yet demanding task in restorative practice. In such situations, the use of an anatomical post plays a vital role in providing adequate retention and stability to the final restoration within the remaining tooth structure [1].

The long-term outcome of root canal-treated teeth largely depends on the amount of residual sound tooth structure. When there is extensive loss of tooth structure, the tooth becomes less capable of withstanding masticatory forces, making it necessary to place a post to retain a core that will replace the lost structure [1].

Various clinical case reports have described direct intraoral techniques for fabricating anatomical post and core restorations. However, these methods are often technique-sensitive, time-consuming, and labor-intensive. Additionally, the handling of composite resin can be challenging due to its sticky and adhesive nature [2].

Therefore, this case report presents a novel indirect extraoral approach for fabricating an anatomical post and core restoration.

## Case presentation

A 22-year-old male patient presented with a chief complaint of a fractured tooth in the upper anterior region for the past four years. He also reported a history of intermittent pain associated with the same tooth, for which root canal treatment had been initiated one year earlier. The pain was described as sudden in onset, intermittent, pricking in nature, non-radiating, aggravated during mastication, and relieved by medication.

**Figure 1:**
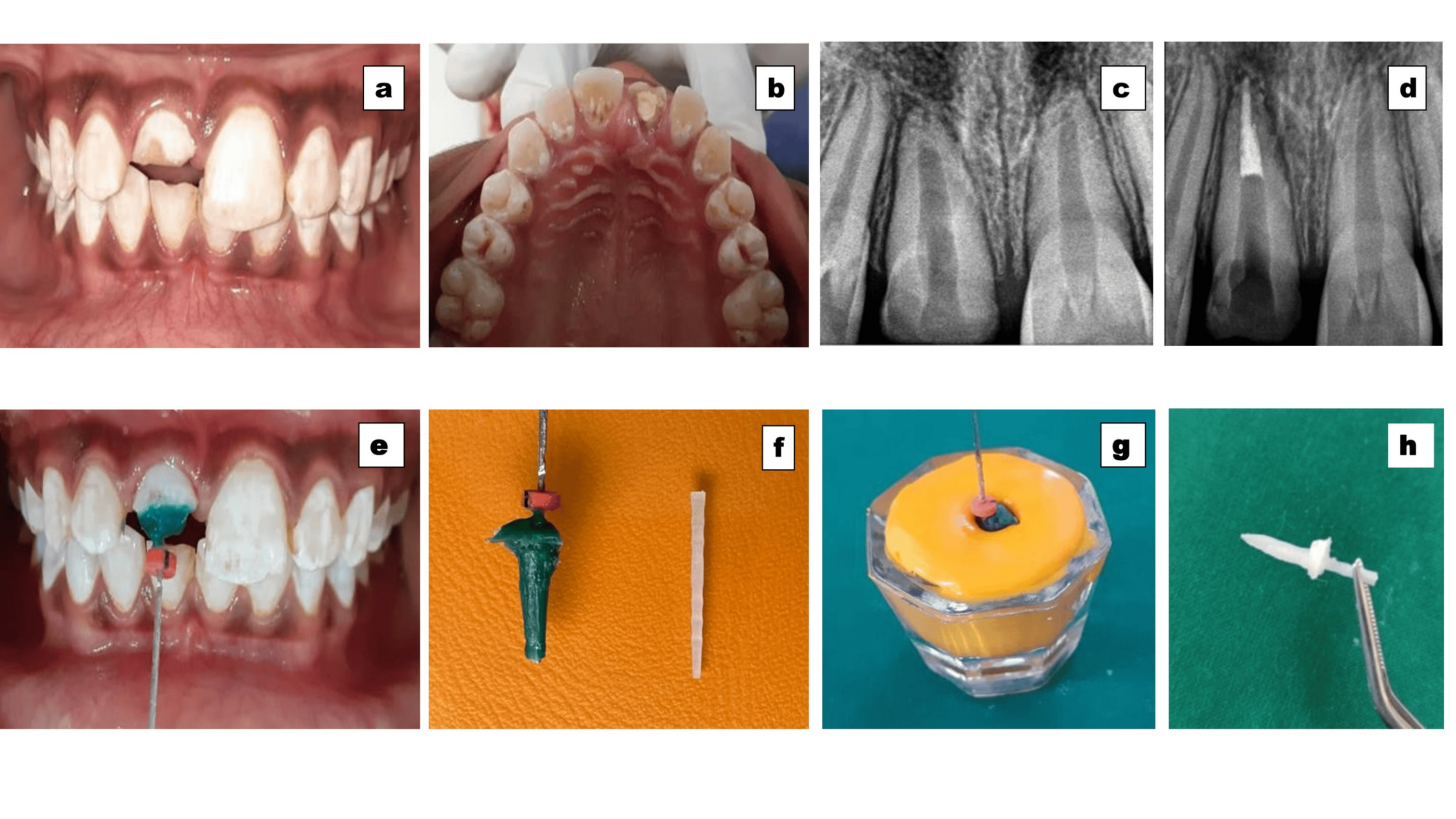
Fabrication of the anatomic post. (a) Preoperative intraoral view showing a fractured maxillary anterior tooth with significant coronal structure loss. (b) Palatal view of maxillary arch illustrating the extent of tooth destruction. (c) Preoperative intraoral periapical radiograph showing root canal condition. (d) Post space preparation was done. (e) An intracanal wax pattern was taken. (f) Comparison of the intracanal wax pattern with a prefabricated fiber post. (g) The putty index used to record the intracanal wax pattern. (h) Final customized anatomical fiber post ready for cementation.

On clinical and radiographic examination, tooth 11 showed incomplete root canal treatment with features suggestive of persistent apical periodontitis (Figures 1A-1C). Based on the clinical findings, rehabilitation using an anatomical post, followed by a porcelain-fused-to-metal crown, was planned to restore both function and esthetics.

All procedures were carried out under strict aseptic conditions with local anesthesia. During the initial visit, the access cavity was refined, and the canal was thoroughly irrigated. Working length was determined, and the canal, initially negotiable with a #70 K-file (MANI Inc., Utsunomiya, Japan), was enlarged up to size #80 (MANI Inc.) using hand instrumentation with a circumferential filing technique under continuous irrigation with 5.25% sodium hypochlorite (Prevest Denpro Limited, Jammu, India). The canal was then dried, and calcium hydroxide was placed as an intracanal medicament. A temporary restoration was used to seal the access cavity.

At the subsequent visit, the patient was asymptomatic. The canal was obturated using gutta-percha along with a resin-based sealer (AH Plus, Dentsply, Charlotte, NC) using the lateral compaction technique. The access cavity was restored with glass ionomer cement, and the patient was kept under observation.

At the one-month follow-up, the patient remained symptom-free, and radiographic findings indicated healing of the periapical lesion. Hence, post placement was planned. Gutta-percha was partially removed using a heated plugger, ensuring that 4 mm of apical seal was maintained. Undercuts within the canal were eliminated using an H-file in a circumferential manner (Figure 1D).

A wax pattern of the canal space was fabricated using inlay wax (Figure 1E). Additional silicone was mixed and placed in a glass dappen dish, and the wax pattern was embedded into the material to create a negative replica of the post space (Figure 1G). After setting, the wax pattern was retrieved.

**Figure 2:**
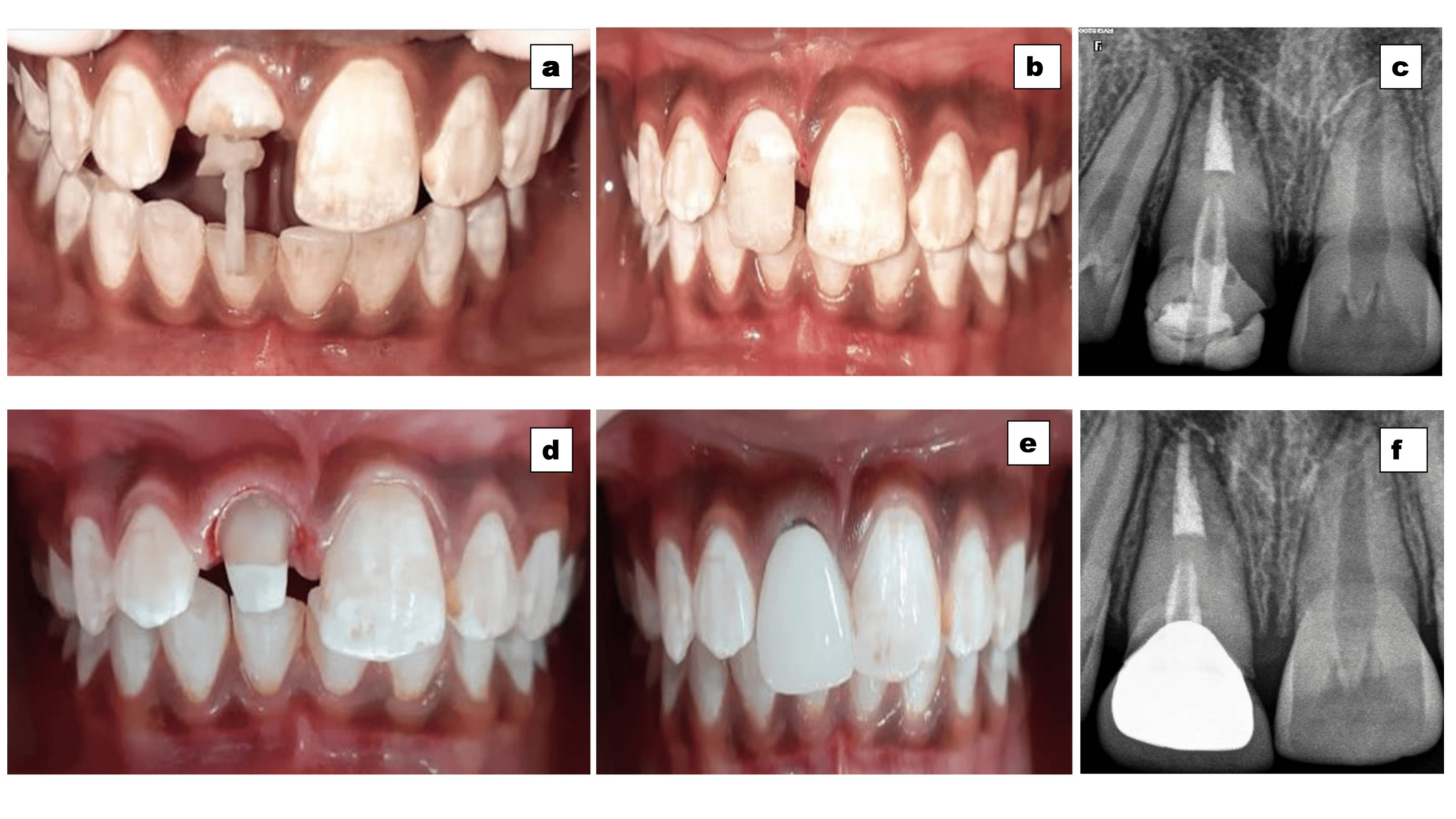
Cementation of the anatomic post. (a) Trial placement of the anatomical post within the canal. (b) Core build-up done. (c) Radiographic confirmation of post placement within the canal. (d) Crown preparation. (e) Final prosthetic restoration of the tooth. (f) Postoperative radiograph.

The fiber post (RelyX Fiber Post, 3M, Saint Paul, MN) was treated with a silane coupling agent, and a nano-hybrid composite resin (Tetric N-Ceram) was adapted over it. This assembly was then inserted into the silicone mold, replicating the canal anatomy (Figure 1H). Initial light curing was carried out for 15 seconds, followed by an additional 15 seconds after removal from the mold. The customized anatomical post was tried intraorally to verify its fit and retention (Figure 2A).

The post was subsequently luted using a dual-cure resin cement (RelyX Unicem 2, 3M, Saint Paul, MN), followed by core build-up (Figures 2B, 2C). Tooth preparation was completed to receive a metal-ceramic crown (Figure 2D). Gingival retraction was performed using a Sure-Endo 00 retraction cord, and an impression was made using additional silicone material. A porcelain-fused-to-metal crown was fabricated and cemented with dual-cure resin cement (Figure 2E). The patient was scheduled for periodic follow-ups to evaluate the success of the treatment (Figure 2F).

## Discussion

The use of an intraradicular post is a well-established method for reinforcing endodontically treated teeth, especially in cases where the remaining coronal structure is inadequate to retain a definitive restoration. Traditionally, custom-cast metal post and core systems have been widely used for teeth with extensive structural loss. These posts, fabricated from direct canal impressions, generally offer precise adaptation, improved retention, and a relatively thin cement layer [3]. However, despite these advantages, cast metal posts are associated with several limitations, including increased treatment time, higher laboratory costs, lack of chemical adhesion to tooth structure, susceptibility to corrosion, and difficulty in retrieval when required. Additionally, their elastic modulus differs significantly from that of dentin. This disparity in stiffness may generate a wedging effect within the root canal, thereby increasing the risk of catastrophic root fractures and ultimately leading to unfavorable outcomes [3]. Earlier, it was commonly believed that post placement inherently strengthened endodontically treated teeth [4]. Nevertheless, current evidence suggests that the preservation of remaining dentin plays a far more critical role in maintaining the structural integrity of the tooth.

Prefabricated fiber posts, although widely used, often fail to adapt adequately to flared or widened root canals. This results in compromised retention and necessitates the use of a thicker luting cement layer to compensate for the mismatch between the post and canal walls [5]. Such increased cement thickness may lead to the incorporation of voids or air bubbles, which can weaken the adhesive interface and predispose the restoration to debonding or failure.

The modification of prefabricated fiber posts into anatomical posts offers a more precise adaptation to the internal canal morphology. This approach ensures a closer fit, reduces the thickness of the luting cement, and promotes a more uniform cement layer, thereby enhancing retention [6].

The anatomical post technique improves the conformity of the post to canal walls while minimizing cement-related discrepancies. Gomes et al. reported that reinforcing the root canal space with composite resin significantly improves fracture resistance in structurally compromised teeth compared to conventional direct anatomical post techniques [7].

Furthermore, several studies have emphasized the importance of achieving a durable adhesive bond between restorative materials and root dentin, particularly in cases involving flared canals. However, the effectiveness of this bond can be influenced by various intrinsic and extrinsic factors. Macedo et al. demonstrated that relining of fiber posts significantly enhances pull-out bond strength when compared to non-relined posts [8]. The advantages of this technique include its elimination of additional laboratory procedures, cost-effectiveness, ease of chairside application, reduced clinical time, and minimal material requirements [9]. With the increasing demand for esthetic and conservative restorative approaches, the use of indirect anatomical posts represents a practical and efficient solution for the management of wide and flared root canals in contemporary clinical practice [10].

## Conclusions

This case demonstrates that a customized anatomical post and core can be used as a practical approach for restoring a severely compromised tooth by improving adaptation to the canal morphology and providing adequate retention.

This technique allowed satisfactory functional rehabilitation and contributed to the structural support of the remaining tooth. Within the limitations of a single case, the outcome suggests that this method may be considered as a viable treatment option in similar clinical situations, although further evidence is required to establish its broader applicability and long-term success.
